# Targeting NK Cell Checkpoint Receptors or Molecules for Cancer Immunotherapy

**DOI:** 10.3389/fimmu.2020.01295

**Published:** 2020-06-23

**Authors:** Cai Zhang, Yuxia Liu

**Affiliations:** Institute of Immunopharmaceutical Sciences, School of Pharmaceutical Sciences, Cheeloo College of Medicine, Shandong University, Jinan, China

**Keywords:** checkpoint receptors, natural killer cells, cancer immunotherapy, checkpoint blockade, tumor microenvironment

## Abstract

Checkpoint blockade therapy, for example using antibodies against CTLA-4 and PD-1/PD-L1, relieves T cells from the suppression by inhibitory checkpoints in the tumor microenvironment; thereby achieving good outcomes in the treatment of different cancer types. Like T cells, natural killer (NK) cell inhibitory receptors function as checkpoints for NK cell activation. Upon interaction with their cognate ligands on infected cells, tumor cells, dendritic cells and regulatory T cells, signals from these receptors severely affect NK cells' activation and effector functions, resulting in NK cell exhaustion. Checkpoint inhibition with antagonistic antibodies (Abs) can rescue NK cell exhaustion and arouse their robust anti-tumor capacity. Most notably, the response to anti-PD-1 therapy can be enhanced by the increased frequency and activation of NK cells, thereby increasing the overall survival of patients with multiple types of cancer. In addition, rescue of NK cell activity could enhance adaptive T cells' anti-tumor activity. Some antagonistic Abs (e.g., anti-TIGIT and anti-NKG2A monoclonal Abs) have extraordinary potential in cancer therapy, as evidenced by their induction of potent anti-tumor immunity through recovering both NK and T cell function. In this review, we summarize the dysfunction of NK cells in the tumor microenvironment and the key NK cell checkpoint receptors or molecules that control NK cell function. We particularly focus on recent advances in the most promising strategies through blockade of NK cell checkpoints or their combination with other approaches to more effectively reject tumors.

## Introduction

In the past few years, cancer immunotherapy has shown great promise. Among the most promising therapeutic modalities, chimeric antigen receptor T cell (CAR-T) and immune checkpoint blockade therapies have demonstrated unprecedented clinical success and represent a turning point in cancer treatment. In particular, checkpoint blockade, such as the use of monoclonal antibodies (mAbs) against cytotoxic T-lymphocyte associated protein 4 (CTLA-4), programmed death ligand 1 (PD-L1), and programmed cell death 1 (PD-1), has shown impressive potential to treat different cancer types by relieving the suppression from the inhibitory checkpoints in the tumor microenvironment (TME) (1, 2). Both CTLA-4 and PD-1 were originally identified as co-inhibitory receptors on T cells; therefore, checkpoint blockade immunotherapy has concentrated on recovering T cell-mediated anti-tumor capacity to regress tumors (2). Antibodies (Abs) against CTLA-4 (Ipilimumab), PD-1 (Pembrolizumab, Nivolumab, and Cemiplimab), and PD-L1 (Durvalumab, Atezolizumab, and Avelumab) have been approved by the Food and Drug Administration (FDA) to treat patients with advanced melanoma, lymphoma, non-small cell lung cancer (NSCLC), renal cell carcinoma (RCC), bladder cancer, head and neck squamous cell cancer (HNSCC), liver cancer, and Merkel cell carcinoma. Currently, mAbs that target other checkpoint receptors, such as T cell immunoglobulin and mucin domain-3 (TIM-3) and lymphocyte-activation gene 3 (LAG-3), are being tested pre-clinically or are in clinical trials (3–5).

Natural killer (NK) cells are the most important effectors of innate immune surveillance because of their ability to kill virus-infected cells and tumor cells without the need for prior activation. NK cells not only recognize and exert direct cytotoxicity against target cells rapidly at the early stage of tumorigenesis, but also engage in cross-talk with immune cells, including dendritic cells (DCs), to alter adaptive immune responses, which enhances anti-tumor responses mediated by CD8^+^T cells. In multiple tumor settings, the antitumor abilities of CD8^+^ T cells require NK cell function (6). Intriguingly, NK cells have adaptive immunity features and can obtain memory-like responses similar to T and B cells (7–9). NK cells express multiple activating receptors, inhibitory receptors, and cytokine or chemokine receptors on their surfaces. NK cell activation and function rely on a balance between signaling from inhibitory receptors and activating receptors. Generally, host homeostasis is maintained by a preponderance of inhibitory signals derived from inhibitory receptors over activation signals (10). Similar to T cells, most of the inhibitory receptors on NK cells act as checkpoints to control NK cell activation (11–13). Increasing data indicate that chronic viral infection or the TME can upregulation killer immunoglobulin-like receptors (KIRs), NKG2A, PD-1, TIM-3, and T cell immunoreceptor with Ig and immunoreceptor tyrosine-based inhibition motif domains (TIGIT) on the surfaces of NK cells. Signals from these inhibitory receptors, generated by interactions with their cognate ligands on tumor cells, infected cells, DCs, and regulatory T cells (Tregs), severely affect the activation and effector function of NK cells, even resulting in NK cell functional exhaustion (14). Checkpoint inhibition with antagonistic Abs can release the inhibitory signals that limit NK cell activity and restore NK cell function. Notably, some antagonistic Abs (e.g., anti-TIGIT and anti-NKG2A mAbs) can induce potent anti-tumor immunity via the recovery of the functions of both NK and T cells, thus revealing extraordinary potential in cancer therapy (6, 15). In this review, we summarize the dysfunction of NK cells in the TME and the key NK cell checkpoint receptors or molecules that control NK cell function. We particularly focus on recent advances in the most promising strategies for therapy, such as NK cell checkpoint blockade or its combination with other approaches to more effectively reject tumors.

## NK Cell Dysfunction in the Tumor Microenvironment

Although NK cells play crucial roles in controlling tumor growth and clearance of viral infection, NK cell function is often impaired significantly during tumor progression and development, and in chronic infection. Numbers of NK cell infiltrating into tumor sites are usually decreased, and NK cell function and activation are severely inhibited. NK cell dysfunction promotes the progression and metastasis of tumors. Decreased NK cell cytolytic activity and cytokine secretion capacity are associated significantly with poor prognosis in various cancers (16, 17). Activating receptors of NK cells, such as NKG2D, CD226, and NKp30, are downregulated on NK cells and correlate inversely with the progression and metastasis of melanoma, colorectal carcinoma, hepatocellular carcinoma (HCC), breast cancer, prostate cancer, gastric cancer, and others (18). Pulmonary or hepatic metastases are accelerated if NK cells are depleted in multiple mouse models (19–21). High numbers of tumor-infiltrating NK cells, high levels of NK activating receptors, and a high capacity for NK lysis correlate positively with longer overall survival and better prognosis (18, 22).

Many factors in the TME prevent NK cells infiltrating inside the tumor sites and hamper their activation and anti-tumor activity (23–25). Extracellular matrix components, such as collagen and hyaluronic acid, are densely packed in pancreatic cancer tissues, which prevent NK cells migrating into the tumor bed (26, 27). Neuroblastoma cells prevent NK cell recruitment by modulating the expression of chemokine receptors on NK cells (25, 28). Stromal cells, tumor-associated macrophages (TAMs), myeloid-derived suppressive cells (MDSCs), and Tregs in the TME inhibit NK cell activation and function by the secretion of immunosuppressive cytokines or interfering with receptor expression of NK cells. Transforming growth factor-β (TGF-β), IL-10, prostaglandin E2 (PEG2), and indoleamine 2,3-dioxygenase (IDO) are the major soluble modulators secreted in the TME, which negatively regulate NK cell function (29, 30). TGF-β inhibits IFN-γ secretion and NK cell cytolytic activity by suppressing the SMAD3 signal pathway. It also silences the expression of NKG2D and NKp30 on NK cells, as well as decreases the expression of NKG2D ligands on tumor cells (31). TGF-β is reported to block IL-15-induced mTOR signaling, thus attenuating the metabolic activity, proliferation, receptor expression, and cytotoxicity of NK cells (32). The TME-derived TGF-β can induce NK cells to transdifferentiate into innate lymphoid cell (ILC) type 1 cells (ILC1), which have no cytotoxic activity, thus promoting tumor escape from immune attack (33). Tregs inhibit the effector function of NK cells by directly secreting TGF-β or downregulating the expression of NKp30 and NKG2D indirectly using membrane-bound TGF-β (34, 35). MDSCs drive the differentiation and expansion of Tregs, thus reducing the anti-tumor effect of both cytotoxic T lymphocytes (CTLs) and NK cells. Membrane-bound TGF-β on MDSCs can also downregulate NK cytokine secretion and cell-mediated cytotoxicity via producing nitric oxide (NO) or in a method dependent on NK-expressed NKp30 (36–38). Recently, NK cell metabolism has been highlighted in determining NK cell fate and facilitating NK cell survival, proliferation, and function. Many factors in the TME modulate NK cell metabolism and contribute to NK cell dysfunction. TGF-β can suppress NK cell metabolism either directly by impairing glycolysis-promoting mTOR signaling, or by inhibiting mitochondrial metabolism via canonical TGF-β signaling (32, 39). Tumor-infiltrating NK cells can upregulate the expression of fructose-1,6-bisphosphatase (FBP1), which impairs glycolysis, thus promoting NK cell dysfunction in the lung cancer microenvironment (40). Insufficient levels of glucose and amino acids (consumed by tumor cells) in the TME can inhibit NK cell metabolism, including oxidative phosphorylation (OXPHOS) and glycolysis. The high levels of metabolic end products in the TME, such as lactate, dampen NK cell metabolism by inducing mitochondrial dysfunction, leading to apoptosis of tumor-infiltrating liver-resident NK cells in patients with colorectal liver metastasis (41). Adenosine, a critical immunosuppressive purine metabolite in the TME, inhibits the activity of tumor-infiltrating immune cells, including T cells, DCs, macrophages and NK cells, and also enhances the immunosuppressive effect of Tregs and MDSCs (42, 43). By binding to purinergic adenosine receptors on surfaces of NK cells, adenosine inhibits the maturation, activation, proliferation, cytokine production and cytolytic capacity of NK cells (44–46).

Tumor cells, antigen-presenting cells (APCs), Tregs, and MDSCs in the TME express high levels of PD-L1 and other ligands of immune checkpoint molecules, which prevent NK cell activation, even resulting in NK cell dysfunction or exhaustion (23). Meanwhile, the immunosuppressive TME alters the balance between inhibitory or activating NK cell receptors by downregulating activating receptor expression and upregulating inhibitory receptor expression, including PD-1, TIM-3, LAG-3, NKG2A, and TIGIT, which are referred to as checkpoint receptors. The interaction between checkpoint receptors and their respective ligands is the major contributor to NK cell exhaustion. Checkpoint blockade can reverse NK cell function in multiple tumors (25). Therefore, NK cell-based immunotherapy targeting checkpoint molecules has shown marked promise to treat both solid tumors and hematological malignancies.

## The Key NK Cell Checkpoint Receptors or Molecules That Control NK Cell Activation and Function

### KIR

Human KIRs are encoded within the leukocyte receptor complex (LRC) on chromosome 19. They belong to the Ig superfamily, and interact with major histocompatibility complex (MHC) class I molecules on target cells. The KIR family comprises both inhibitory and activating receptors. In the inhibitory KIRs, inhibitory signaling is transduced via the immunoreceptor tyrosine-based inhibition motifs (ITIMs) located in the long cytoplasmic tails. The activating KIRs are short-tail and are associated with the adaptor molecule DAP12 or FcγR to provide activating signals to NK cells (47). Each inhibitory KIR contains two (KIR2DL) or three (KIR3DL) extracellular Ig domains which confer their specificity for human leukocyte antigen (HLA) molecules. Thus, in this way, they can sense the downregulation or alteration of HLA molecules during viral infection and malignancy (48). Generally, KIRs are constitutively expressed on NK cells, and the inhibitory signals from inhibitory KIRs typically predominate over activating signals from activating KIRs. Thus, the activation and function of NK cells are fine-tuned via inhibitory KIRs to prevent damage to normal healthy cells mediated by NK cells and the induction of virus infected cells or tumor cells.

However, during tumor malignancy, inhibitory KIRs are upregulated whereas activating KIRs are downregulated in various types of cancer, such as lymphoma, leukemia, breast cancer, NSCLC, skin cancer, and biliary cancer (14, 49). These alterations restrain the activation and anti-tumor capacity of NK cells, thus contributing to tumor escape from immunosurveillance. Inhibitory KIRs were the first identified NK cell checkpoint receptors (50). mAbs recognizing inhibitory KIRs might induce NK cells' anti-tumor activity by blockading the inhibitory signaling pathway, thus several anti-KIR mAbs, for example those targeting KIR2DL1-3, have been applied to treat patients with leukemia, lymphoma, multiple myeloma, and some solid tumors in multiple clinical trials, which have been demonstrated as safe with limited side effects (14, 51).

### NKG2A

NKG2A, a C-type lectin family member, is a type II membrane receptor encoded by a gene on chromosome 12 within the NK gene complex. CD94, lacking an intracellular signal domain, is covalently associated with NKG2A, which has ITIM domains in its cytoplasmic tail, to form a CD94/NKG2A heterodimer. NKG2A is expressed on approximately 50% of NK cells and 5% of CD8^+^T cells in human peripheral blood. Cytokines, such as IL-15, and chronic inflammation, upregulate NKG2A expression. Through ligation with its ligand HLA-E in humans or Qa-1 in mice, NKG2A transduces negative signaling to NK cells to prevent NK cell activation and thus suppress cytokine secretion and cytotoxicity. Notably, the surface expression of HLA-E depends on peptides derived from the conserved region of the leader peptide sequences of HLA-A, HLA-B, HLA-C, and HLA-G. Therefore, the CD94-NKG2A axis can monitor the antigen presenting capacity and the expression of HLA class I molecules on target cells (48). Similar to KIRs, the ITIM-based inhibitory signal from CD94/NKG2A is dominant over the activation signaling from its counterpart CD94/NKG2C.

Tumor-infiltrating NK cells express high levels of NKG2A in comparison with peripheral or splenic NK cells, while HLA-E is upregulated on both hematopoietic and solid tumors (17, 52). The interaction of NKG2A and HLA-E inhibited the anti-tumor effector function of both NK cells and some CD8^+^T cell subsets that upregulate NKG2A in the TME. In patients with HCC, high levels of NKG2A on tumor-infiltrating NK cells and high levels of HLA-E in intratumor tissues were observed, and poor prognosis was associated with the functional exhaustion of high NKG2A-expressing CD56^dim^ NK cells (17). Worse survival was associated with a high density of NKG2A^+^ tumor infiltrating NK cells and high levels of HLA-E in tumor tissues in patients with ovarian cancer, RCC, colorectal cancer, breast cancer, and lung cancer (51–53). NKG2A is regarded as the major immune checkpoint of NK cells because of the suppressive effect of NKG2A on NK cells. The use of anti-NKG2A mAbs for therapeutic blockade of NKG2A restored the cytolytic activity of NK cells against HLA-E^+^ chronic lymphocytic leukemia (CLL) targets, thus improving NK cell dysfunction (54). Increasing evidence indicates that interruption of the interaction between NKG2A and HLA-E can induce an effective anti-tumor immune response. NKG2A blockade enhanced both CD8^+^T cell and NK cell-mediated anti-tumor effects. In particular, combined co-blockade with an anti-PD-L1 mAb promoted the generation of protective anti-tumor memory in various tumors, including lymphoma in a mouse model, HNSCC patients, and CRC patients (15). mAbs targeting NKG2A, alone or combined with other therapeutic strategies, are being used currently to restore the effector function of NK and CD8^+^T cells in clinical trials (15).

### TIGIT and CD96

TIGIT, a member of immunoglobulin (Ig) superfamily, consists of an extracellular Ig V domain, a transmembrane domain, and a cytoplasmic tail containing an ITIM motif and an Ig tail-tyrosine (ITT)-like motif, which transduces inhibitory signals. CD96 is also an Ig superfamily member and comprises three extracellular Ig-like domains and a short ITIM motif-containing cytoplasmic tail, mediating inhibitory functions. TIGIT and CD96 act as inhibitory receptors and compete with activating receptor DNAX accessory molecule-1 (DNAM-1) or CD226 to bind to CD155 and CD112. The affinity of TIGIT or CD96 binding to CD155 is higher than that to DNAM-1; therefore, similar to other NK cell inhibitory receptors, inhibitory signals from TIGIT or CD96 are usually dominant over the signals from DNAM-1-mediated co-stimulation. Human NK cells, Tregs, memory T cells, and some CD8^+^T cells, express TIGIT, while in the TME, tumor-infiltrating NK cells show upregulated TIGIT expression accompanied by decreased DNAM-1 levels. CD155 is barely expressed in healthy human tissues, but it is dramatically overexpressed in various cancers, being considered an independent prognostic marker for poor prognosis for patients with breast cancer (55). Binding of TIGIT with CD155 suppresses the NK cells' cytotoxicity and IFN-γ production, whereas blockade using an anti-TIGIT antibody could reverse this effect (56). TIGIT was reported to be the key checkpoint receptor associated with NK cell dysfunction and exhaustion in several tumor-bearing mouse models (including colon cancer, melanoma, breast cancer, and fibrosarcoma) and in patients with CRC. Blockade of TIGIT recovered the NK cell function and anti-tumor effector activity, improved memory responses after tumor rechallenge, and elicited tumor-specific T cell immune responses, which were dependent on the presence of NK cells (6). CD96's interaction with CD155 resulted in reduced IFN-γ production but not NK cell cytolysis. Blockade of CD96 or *CD96*-/- could enhance NK cell's capacity to produce IFN-γ and led to improved control of lung metastasis; these anti-metastatic effects depended on IFN-γ, CD226, and NK cells (57–59). Numbers of CD96^+^ NK cells were increased in tumor sites in HCC and exhibited functional exhaustion with decreased of IFN-γ and TNF-α production, low perforin and granzyme B levels, and high IL-10 and TGF-β expression. Moreover, the high CD96 levels in tumor tissues correlated with the shorter overall survival and disease-free survival (60). It was proposed that TIGIT and CD96 play complementary roles in the control of NK cell effector function (51). Therefore, TIGIT, CD96, and CD155 are deemed as key immune checkpoints for NK cells, and the blockade of these molecules has shown great promise in tumor immunotherapy (61–64). TIGIT is often coexpressed with PD-1; therefore, combination blockade of TIGIT and PD-1 could reverse functional exhaustion more effectively, as displayed in patients with advanced melanoma, who showed increased expansion and an elicited anti-tumor immune response (65).

### PD-1

PD-1 is primarily induced in activated T cells, it is also expressed on NK cells, NKT cells, B cells, and myeloid cells. The PD-1^+^NK cell subset constitutes 25% of NK cells in healthy donors, it is confined to CD56^dim^ mature NK cells (66). The proportions of PD-1^+^ NK subset and PD-1 expression on NK cells are increased in the TME of various cancers, including colon cancer, liver cancer, gastric cancer, esophageal cancer, ovarian cancer, head and neck cancer, and Hodgkin lymphoma, where its ligands, PD-L1 or PD-L2, are expressed at high level (67). The interaction between PD-1 and its ligands provides negative signals for NK cell activation, and inhibits the anti-tumor capacity of NK cells (67, 68). PD-1^+^NK cells exhibit impaired cytokine production, decreased cytolytic activity, and poor proliferation, even becoming functionally exhausted (69). There is an association between PD-1^+^NK cell levels and poor outcome. Blocking the PD-1/PD-L1 interaction with anti-PD-1 or PD-L1 antibodies could reverse the dysfunctional status of PD-1^+^NK cells, which manifests as significantly augmented production of cytokines, NK cell cytotoxicity, and marked suppression of tumor growth *in vivo* (67, 69–71). Therefore, clinically, PD-1 blockade not only unleashes T cells to attack tumor cells, but also restores the anti-tumor responses of NK cells. Notably, the enhancement of NK cell anti-tumor efficacy by blockade of PD-1/PD-L1 is more important for the treatment of patients with tumors that are defective in MHC class I expression or display low mutational loads, because T cells are often inactive in these settings. Indeed, most Hodgkin's lymphomas express decreased or negative MHC class I molecules but show upregulated PD-L1 expression, yet patients responded well to immunotherapy blockading PD-1/PD-L1, indicating the pivotal role of the anti-tumor efficacy of NK cells (70, 72).

### TIM-3

TIM-3 is a type I transmembrane protein belonging to the Ig superfamily, expressed on CD4^+^T, CD8^+^T, Treg, NK, NKT and myeloid cells. TIM-3 ligands include phosphatidylserine (PtdSer), carcinoembryonic antigen cell adhesion molecule 1 (CEACAM-1), high mobility group protein B1 proteins (HMGB1), and galectin-9. The cytoplasmic tail of TIM-3 does not have an ITIM motif but comprises five conserved tyrosine residues that are important for TIM-3 signal transduction. Upon binding of TIM-3 with its ligands, the tyrosine residues recruit certain signaling components that transduce inhibitory signaling, thereby promoting the inhibition, anergy, or exhaustion of immune cells (51, 73). TIM-3 has been regarded as an activation or maturation marker on NK cells, because it induces IFN-γ production and promotes NK cell maturation at the early stage upon engagement with its ligand galectin-9 (74, 75). However, persistently high expression of TIM-3 contributes to NK cell dysfunction and exhaustion. TIM-3 is highly expressed on peripheral NK cells from patients with various types of solid tumors, such as lung cancer, gastric cancer, and advanced melanoma, and correlates with NK cell dysfunction and exhaustion (76–78). Tumor-infiltrating NK cells in particular show upregulated TIM-3 expression, which can predict poor prognosis in patients with liver cancer, NSCLC, endometrial cancer, and other types of tumors (79–81). Both conventional NK cells and liver-resident NK cells from patients with liver cancer express high levels of TIM-3, accompanied by decreased capacity of cytokine production and cytotoxicity (79). The percentages of tumor-infiltrating TIM-3^+^ NK cells correlated negatively with the survival of patients with HCC. TIM-3 blockade significantly restored IFN-γ production, cytotoxicity, and proliferation of both liver-resident NK and conventional NK cells. Mechanistically, the binding of the endogenous ligand PtdSer with TIM-3 induced the dysregulation of NK cells through interrupting the PI3K/mTORC1/p-S6 signaling pathway. Importantly, TIM-3 knockdown or antibody blockade reduced tumor growth and prolonged the overall survival of orthotopical liver tumor-bearing mice in an NK cell-dependent manner (79). TNF-α was reported to induce NK cell expression of TIM-3 and NK cell dysfunction via the NF-κB pathway. Tumor invasion, lymph node metastasis, and poor staging in patients with esophageal cancer was associated with high levels of TIM-3 on tumor-infiltrating NK cells (80). The high levels of TIM-3 on tumor-infiltrating NK cells hampered the functional potential of NK cells after stimulation with IL-2/IL-15/IL-21 (82). In addition, MHC class I-deficient tumor cells led to selective upregulation of TIM-3 and PD-1 expression on intratumoral NK cells, which showed an exhausted phenotype and dramatically reduced cytotoxicity and IFN-γ production. IL-21 could reverse the functions of exhausted TIM-3^+^PD-1^+^ NK cells by activating the STAT1 and PI3K-AKT-Foxo1 signaling pathways (83). Furthermore, TIM-3 and PD-1 blockade combined with IL-21 revived the anti-tumor effects of exhausted NK cells in patients with advanced MHC class I-deficient tumors (84).

### LAG-3

LAG-3 is a member of the Ig superfamily of receptors and acts as an inhibitory receptor. LAG-3 expressed on plasmacytoid dendritic cells (pDCs), B cells, NK cells, and activated T cells. Its ligands include LSECtin, a member of the DC-SIGN family, and MHC class II molecules. LAG-3 is expressed in liver cancer and several other tumors (85). Recently, fibrinogen-like protein 1 (FGL1) was identified as another MHC II-independent functional LAG-3 ligand. FGL1 shows high expression on human cancer cells, and inhibits the antigen-specific T cell response, while blockade of the LAG-3–FGL1 interaction using antibodies enhanced its anti-tumor effects (86). LAG-3, PD-1 and other inhibitory receptors are often co-expressed on tumor-infiltrating T cells in various types of cancers, such as squamous cell carcinoma, NSCLC, ovarian cancer, melanoma, colorectal adenocarcinoma, and fibrosarcoma (87–90). Co-blockade of LAG-3 and PD-1 could reverse T cell exhaustion and disrupt tumor growth more effectively than single antibody treatment, particularly for tumors that are resistant to singe antibody therapy (91, 92). Although blockade of LAG-3 alone seemed to have no effect on NK cell lysis against various targets, several reports showed that chronic stimulation of NK cells could increase LAG-3 surface expression and that of other co-inhibitory receptors, which contributed to NK cell exhaustion and weakened the anti-tumor activity of NK cells. In this situation, blocking LAG-3 could restore NK cells' cytotoxic capacity (93, 94). In a 4T1 mouse lung metastatic breast cancer model, lung infiltrating NK cells manifested a dysfunctional state, with impaired lytic activity and low capacity to produce IFN-γ, concomitant with increased expression of inhibitory receptors and the loss of activating receptors. IL-12 treatment could rescue the NK cell cytotoxicity, but also upregulated the levels of coinhibitory checkpoints such as LAG-3, PD-1, and TIGIT, thereby restraining the NK cell-mediated anti-metastatic effect. Remarkably, a synergistic effect of IL-12 therapy combined with checkpoint blockade using anti-LAG-3 and PD-1 antibodies markedly reactivated and amplified the antitumor capacity on NK cells, leading to a forceful control of metastasis by NK cells (95).

### Adenosine Pathway

Extracellular adenosine is a nucleoside and derivative of ATP. The levels of ATP and adenosine are highly elevated in the TME in response to hypoxia, apoptosis, and inflammation (42, 96). ATP catabolism in tumors is primarily mediated by ecto-nucleotidases CD39 and 5′-nucleotidase CD73, both of which are over-expressed on various types of cells, including tumor cells, stromal cells, Tregs, MDSCs, and T cells in the TME (42, 97). CD39 converts ATP or ADP to ADP or AMP, respectively; while CD73 dephosphorylates AMP to adenosine. The high levels of adenosine impair the function of T cells, DCs, macrophages and NK cells by binding to adenosine receptors, primarily A2A receptor (A2AR) (42). Tumor-infiltrating NK cells can obtain CD73 expression and play immunosuppressive role by producing adenosine (98). The accumulation of adenosine in TME strongly inhibits the anti-tumor efficacy of NK cells, including suppressing the maturation, activation, cytokine production and cytotoxicity by binding to A2ARs on the surface of NK cells. Accordingly, adenosine pathway is considered as a key checkpoint for both T cells and NK cells. Blockade of adenosine pathway with A2A receptor inhibitor or antagonist, or anti-CD73 mAb can reduce the tumor growth and metastasis, and prolong survival in several types of tumors (99–102).

### IL-1R8

IL-1R8, also known as single immunoglobulin IL-1R-related receptor, SIGIRR, comprises a single extracellular domain, a transmembrane domain, a cytoplasmic TIR domain, and a long tail. It commonly functions to negatively regulate ILR and TLR signaling, therefore modulating innate and adaptive immune responses (103). Recently, IL-1R8 was identified as a checkpoint molecule for NK cells by virtue of its regulation of NK cell maturation and function (104). Although IL-1R8 is expressed in many cells (such as tumor cells, epithelial cells, and leukocytes), it is expressed at high level in NK cells, and is particularly upregulated in mature NK cells. IL-1R8 can directly regulate the IL-18 signaling pathway, thus impairing NK cell activation. IL-1R8 deficiency in NK cells enhanced the IL-18-mediated activation of JNK and mTOR pathways, which are important for the metabolism, differentiation, and activation of NK cells (104). Genetic blockade of IL-1R8 restored NK cell-mediated resistance to diethylnitrosamine (DEN)-induced liver carcinogenesis, colon cancer-derived liver metastasis and mucinous carcinoma associated antigen (MCA)-induced lung metastasis (104). Similarly, IL-1R8 deficiency unleased NK cell-mediated control of MCMV infection, which depends on increased NK cell cytotoxicity and production of IFN-γ (104). Thus, IL-1R8 blockade might be a novel therapeutic strategy to recover the anti-tumor and anti-viral efficacy of NK cells.

### CIS

Cytokine-inducible Src-homology-2 containing protein (CIS), a member of the suppressor-of-cytokine-signaling (SOCS) family, is regarded as a potent intracellular NK cell checkpoint molecule in the anti-tumor immune response (105). In NK cells, CIS plays a vital role by negatively regulating IL-15 signaling. CIS depletion improved NK cells' response to IL-15, as evidenced by increased proliferation, survival, IFN-γ production, and cytolytic activity against tumors. The molecular mechanism revealed that CIS can directly target JAK1, a downstream protein of IL-15, by inhibiting the kinase activity of JAK and reducing the JAK protein level through ubiquitination and proteasomal degradation. Moreover, CIS-deficient NK cells protect mice by making them resistant to lung metastasis of melanoma, breast cancer, and prostate cancer (105). Further study showed that targeted deletion of CIS can lower the threshold of NK cell activation, thus decreasing the requirement for priming and enhancing NK cells' anti-tumor function (106). Accordingly, CIS is considered as a potent intracellular NK cell checkpoint. Ablation of CIS or blockade of the CIS-JAK pathway might unleash NK cell anti-tumor efficacy, and thus might become a novel therapeutic strategy for tumor immunotherapy, especially for the prevention of tumor metastasis.

### E3 Ubiquitin Ligase CbL-b and TRIM29

E3 ubiquitin ligase Cbl-b (casitas B-lineage lymphoma-b) was reported to regulate cancer metastasis by suppressing NK cell function. Cbl-b depletion or inhibition of its ubiquitylation targets comprising TAM (Tyro3, Axl and Mer) tyrosine kinase receptors efficiently released the anti-metastatic activity of NK cells, as evidenced by reduced metastasis in various mouse tumor models, including mammary cancer and melanoma, dependent on NK cell activation (107). E3 ubiquitin ligase TRIM29 was recently identified as a checkpoint regulator of NK cell function. TRIM29 is upregulated in IL-12^+^IL-18^−^ activated NK cells, and TRIM29 levels correlated negatively with IFN-γ production of NK cells. Conditional deletion of TRIM29 in NK cells increased the production of IFN-γ by NK cells and promoted host defense against viral infection (108). These findings suggest the importance of E3 ubiquitin ligase in the regulation of NK cell function, and some of the molecules, such as Cbl-b and TRIM29, might function as key checkpoint regulators of NK cell activity with important clinical implications.

## Blockade of NK Cell Checkpoints to Rescue NK Cell Dysfunction in Cancer Immunotherapy

### KIR Blockade-Based Cancer Immunotherapy

KIRs were the first discovered NK cell checkpoint receptor in the early 1990s. The first-in-class humanized IgG4 mAb IPH2101, targeting an epitope shared by KIR2D, was produced to block the binding of HLA-C to KIR2D to release NK cell inhibition and enhance NK cells' capacity to kill tumor targets. IPH2101, alone or in combination with Lenalidomide, has been used in phase I/II clinical trials to treat patients with multiple myeloma and acute myeloid leukemia, and have shown good safety and efficacy (NCT00552396, NCT00999830, NCT01222286, NCT01217203, NCT01248455, NCT01256073) (109). The second generation anti-KIR antibody IPH2102 (Lirilumab), a fully human IgG4, has been developed to treat patients with hematological malignancies and solid tumors. Clinical trials have shown evidence that IPH2102 is well-tolerated and had good efficacy (NCT01687387). Phase I/II clinical trials of the combination of PH2102 plus anti-CTLA4 mAb (Ipilimumab) or anti-PD-1 mAb (Nivolumab), or PH2102 plus Nivolumab and Ipilimumab are completed and displayed good safety, tolerability and efficacy in therapy of advanced solid tumors (NCT01750580, NCT01714739). The safety, tolerability, and efficacy assessment of IPH2102 combined with Nivolumab or IDO inhibitor (Epacadostat) is ongoing in the treatment of patients with advanced or metastatic malignancy (NCT03203876, NCT03341936, NCT03347123). IPH4102, a first-in-class humanized mAb selectively targeting KIR3DL2, is currently being applied in a phase I clinical trial to treat relapsed or refractory cutaneous T cell lymphoma (NCT02593045), which has shown good tolerance and encouraging clinical efficacy (110, 111). A multi-cohort phase II trial is ongoing to further confirm the clinical activity, safety, and effect of IPH4102 in other subtypes of KIR3DL2^+^T cell lymphoma (NCT03902184).

### NKG2A Blockade-Based Cancer Immunotherapy

Convincing evidence indicates that blockade of NKG2A can recover the anti-tumor function of tumor-infiltrating NK and CD8^+^T cells. A first-in-class humanized IgG4 mAb against CD94/NKG2A, IPH2201 (Monalizumab), has been employed in phase I trials for therapy of gynecological cancer (NCT02459301) and hematological malignancy after allogenic stem cell transplantation (NCT02921685), which is suggested to be safe, tolerable, and effective. Monalizumab in combination with other Abs or therapeutic agents has shown more promising results. Monalizumab treatment can restore CD107 and IFN-γ production in NK cells against various tumor cells, whereas the combination of monalizumab and anti-PD-L1 mAb (Durvalumab) showed additive effects, as demonstrated by rescue of the anti-tumor activity of both CD8^+^T and NK cells. Monalizumab combined with the anti-EGFR Ab cetuximab enhanced NK cell-mediated antibody-dependent cell-mediated cytotoxicity (ADCC), thus amplifying anti-cancer capacity against Ab-coated tumor cells (15). The phase II clinical trial NCT02643550 is ongoing to assess the safety and validity of monalizumab combined with cetuximab to treat patients who have received prior systemic regimens for recurrent and/or metastatic squamous cell carcinoma of the head and neck (SCCHN). The combined therapy was well-tolerated, and yielded an encouraging outcome comprising an overall response rate (ORR) of 27.5% as compared to the efficacy of ORR of 13% when given cetuximab alone (15, 112). Another phase II clinical trial applying a combination of monolizumab and Durvalumab to treat patients with recurrent or metastatic CRC has just been completed (NCT02671435). The preliminary results indicate encouraging activity with well-tolerated toxicity in patients with microsatellite-stable CRC (MSS-CRC) who were non-responsive to checkpoint blockade for PD-1/PD-L1 (113). Clinical trials assessing the safety and efficacy of anti-NKG2A mAb combined with other agents, such as the BTK inhibitor ibrutinib, are currently ongoing (NCT02557516). A recent interesting study developed NKG2A protein expression blockers (PEBLs) containing an anti-NKG2A ScFv antibody fragment linked to endoplasmic reticulum (ER)-retention domains that set traps to prevent NKG2A transport to the NK cell-surface. Notably, the PEBL-transduced NKG2A^null^ NK cells displayed a more effective antitumor capacity against HLA-E^+^ tumor cells *in vivo* than blockade with anti-NKG2A mAb (114, 115). These impressive results suggested that the adoptive transfer of NKG2A^null^ NK cells could represent a promising therapeutic strategy to treat HLA-E-expression tumors.

### TIGIT Blockade-Based Cancer Immunotherapy

Increasing evidence has shown that the TIGIT/CD96/DNAM-1/CD155 axis controls T cell and NK cell activation and effector function. CD96 and TIGIT-derived inhibitory signals counteract the activating signals from DNAM-1 to form key checkpoints for both CD8^+^T and NK cells. CD155, CD96, or TIGIT blockade is able to reverse the dysfunction of T cells and NK cells in the TME in many types of cancers.

TIGIT is preferentially expressed on CD16^+^NK cells, and blockade of TIGIT significantly augmented trastuzumab-triggered NK cell-mediated anti-tumor immune responses against breast cancer (116). *In vivo* blockade of TIGIT or CD96 using Abs inhibited tumor growth and metastasis in an NK cell-dependent manner and thus improved the overall survival in melanoma and experimental or spontaneous lung metastasis models (6, 58). TIGIT knockout or Ab blockade led to increased IFN-production and enhanced CD8^+^T cells' and NK cells' anti-tumor effects, resulting in colon cancer growth inhibition (117). Combined blockade of PD-1/PD-L1 and TIGIT exhibited a more powerful anti-tumor effect in colon cancer, melanoma, and glioblastoma (GBM) compared with that of monotherapy (6, 65, 118, 119). Currently, phase I and phase II clinical trials using anti-TIGIT mAbs, alone or combined with anti-PD-1/PD-L1 mAbs, are ongoing to evaluate the safety and efficacy in patients with metastatic or locally advanced solid tumors, such as RCC, NSCLC, SCCHN, breast cancer, melanoma, and CRC (NCT02794571, NCT03119428, NCT03563716, NCT03628677, NCT04047862). A phase I/II randomized clinical trial with anti-TIGIT and anti-LAG-3 mAbs, alone or combined with pomalidomide and dexamethasone is recruiting patients with relapsed refractory multiple myeloma who have relapsed after treatment with prior therapies (NCT04150965). A phase III clinical trial is ongoing to evaluate the efficacy of anti-TIGIT mAb (tiragolumab) plus anti-PD-L1 mAb (atezolizumab) and carboplatin and etoposide in patients with chemotherapy-naive extensive-stage small cell lung cancer (NCT04256421). Another phase III clinical trial is about to begin to assess the safety and scientific validity of anti-TIGIT antibody (tiragolumab) combined with atezolizumab in patients with previously untreated locally advanced unresectable or metastatic PD-L1-selected NSCLC (NCT04294810).

### TIM-3 Blockade-Based Cancer Immunotherapy

TIM-3 has gained prominence as a checkpoint receptor and as a potential candidate for cancer immunotherapy. TIM-3 and PD-1 are often co-expressed on tumor-infiltrating T and NK cells, and synergistically mediate immune cell exhaustion (83, 120). Upregulation of TIM-3 correlated with resistance to PD-1 blockade therapy (121–123). TIM-3 blockade *in vivo*, particularly combined with PD-1 blockade, has shown encouraging results, as evidenced by restoration of T and NK cell effector function, and a significant reduction of tumor growth in various preclinical cancer models and clinical trials (78, 121). In some preclinical models, such as sarcoma, prostate cancer, and colon carcinoma, TIM-3 blockade alone appeared to be effective. The co-blockade of TIM-3 and PD-1 displayed increased anti-tumor efficacy, with more complete tumor regression and longer survival, in models of melanoma, fibrosarcoma, colon cancer and leukemia (124–126). NK cells from patients with advanced melanoma expressed high levels of TIM-3 and displayed an exhausted phenotype and function. The high levels of TIM-3 on NK cells correlated with the advance stage and poor prognosis of patients with melanoma. Moreover, TIM-3 blockade could induce the internalization of cell surface TIM-3 and upregulated the expression of IL-2R, thereby reversing the dysfunction and exhaustion of NK cells from patients with metastatic melanoma (78).

Currently, clinical trials (phase I and II) to evaluate the safety and efficacy of anti-TIM-3 antibodies alone or in combination with anti-PD-1/PD-L1 antibodies in patients with advanced solid tumors, lymphoma and leukemia are ongoing. A multicenter, open-label, first-in-human phase I study using anti-TIM-3 antibody (TSR-022) as a monotherapy and in combination with an anti-PD-1 antibodies (TSR-042) or anti-LAG-3 antibodies (TSR-033) is recruiting patients with advanced solid tumors (NCT02817633). A phase II clinical trial (NCT03680508) to study the safety and validity of TSR-042 and TSR-022 during the therapy of patients with primary, locally advanced or metastatic liver cancer has begun to recruit patients. Another phase I clinical trial (NCT03099109) is studying the safety and efficacy of anti-TIM-3 antibody (LY3321367) alone, or combined with anti-PD-L1 antibodies (LY3300054), to treat patients with advanced relapsed/refractory solid tumors. An open-label, multicenter, non-randomized phase I and phase II clinical trial using a humanized mAb against TIM-3 (BGB-A425) combined with humanized mAb against PD-1 (tislelizumab) is recruiting to test the safety and anti-tumor effect in patients with advanced solid tumors (NCT03744468). Novartis Pharmaceuticals are recruiting patients for phase I/II clinical trials to characterize the efficacy, tolerability, and safety of the anti-TIM-3 antibody MGB453 as a single agent or combined with anti-PD-1 antibody (PDR001) and/or chemotherapy drugs to treat patients with acute myeloid leukemia (AML), high risk myelodysplastic syndrome (MDS), or advanced solid tumors (NCT03066648, NCT02608268, NCT03946670). A phase II clinical trial has been initiated to evaluate the safety and efficacy of MBG453 in combination with azacitidine and the BCL2 inhibitor venetoclax to treat patients with AML (NCT04150029). A phase III multi-center, randomized study of MBG453 or placebo added to azacitidine will be recruiting to treat patients with intermediate, high, or very high risk MDS or chronic myelomonocytic leukemia (NCT04266301).

### LAG-3 Blockade-Based Cancer Immunotherapy

As one of the key checkpoint receptors for both T and NK cells, LAG-3 is becoming a major target for blockade to restore the anti-tumor capacity in the therapy of various types of cancer. To date, multiple clinical trials are evaluating several anti-LAG-3 mAbs for both solid and hematological cancers, both alone and in combination with anti-PD-1 mAbs (NCT01968109, NCT02061761, NCT02658981, NCT02966548, NCT03005782, NCT03250832, NCT03311412, NCT03365791, NCT03459222, NCT03489369, NCT03623854, NCT03743766, and NCT04080804). Clinical trials applying anti-LAG-3 mAbs in combination with other checkpoint inhibitors, such as anti-TIM-3 mAbs or anti-CTLA-4 mAbs are also recruiting patients to evaluate the safety and scientific validity of their use to treat patients with advanced solid tumors (NCT02817633, NCT03459222). Some of these clinical trials have indicated promising results, in which the combination of anti-PD-1 and anti-LAG-3 blockade has a synergistic anti-tumor effect compared with monotherapy, and could improve the efficacy in patients who are resistant to anti-PD-1 therapy (3, 127).

### PD-1 Blockade-Based Cancer Immunotherapy

Antibodies targeting PD-L1 and PD-1 have been approved to treat solid and hematological malignancies, including melanoma, bladder cancer, head and neck cancer, RCC, NSCLC, HCC, and other tumors. Although it is assumed that these checkpoint blockade therapies primarily rescue T cell's anti-tumor efficacy, increasing evidence suggests that NK cells potentially respond to PD-1/PD-L1 blockade, particularly for MHC I-deficient tumors, as evidenced by attenuated anti-tumor efficacy after depletion of NK cells (70). A phase II clinical trial has enrolled patients to assess the efficacy of the anti-PD-1 mAb, Pembrolizumab, in terms of the function and exhaustion of NK cells in patients with unresectable stage III or IV melanoma (NCT03241927). Accordingly, blockade of PD-L1/PD-1 could rescue NK and T cell-mediated anti-tumor activity. Notably, the mobilization and re-activation of NK cells using PD-1 blockade is critically important for therapy of patients with HLA^neg^ tumors (e.g., Hodgkin lymphoma) that are resistant to T cell-mediated therapy. Innate and adaptive immune responses may be activated simultaneously by combined blockade of PD-1 and other checkpoints. Currently, multiple clinical trials are ongoing utilizing anti-PD1/PD-L1 antibodies, alone or in combination with other checkpoint blockade antibodies, antiangiogenic bevacizumab, or chemotherapy to treat solid and hematological tumors.

### Blockade of Adenosine Pathway in Cancer Immunotherapy

Targeting adenosine pathway, either blockade CD39 and CD73 with respective mAb to prevent the adenosine production or blockade the A2AR with inhibitors to interrupt adenosine-induced signal pathway, has been evaluated for cancer immunotherapy in pre-clinical studies and clinical trials. Both CD73-deficient mice and administration of anti-CD73 mAbs have displayed significant reduction of tumor growth and metastasis in several types of tumor models (99, 101, 102, 128). Anti-CD73 mAbs (Oleculumab, NZV930) are currently evaluated alone or in combination with other therapies (such as anti-PD-1 mAb) for the treatment of a variety of solid tumors (Table 1). Combination therapy with oleculumab and PD-L1 blockade in patients with pancreatic cancer and MSS-CRC revealed partial responses in 2 out of 20 and 1 out of 21 patients, respectively. Similarly, genetic deletion of A2AR or given A2AR antagonists or inhibitors have shown reduced tumor growth and metastasis, leading to prolonged survival (129–131). Moreover, treatment with A2AR antagonists can enhance the infiltration of CD8^+^T cells and NK cells into tumor sites, while simultaneously reduce the frequency of Tregs (132, 133). Currently, several A2AR antagonists (such as CPI-444, AZD4635, NIR178, and PBF-509) are being evaluated as single agent or in combination with anti-PD-1 mAb in clinical trials for treatment of cancer patients bearing solid tumors (Table 1). Combinations of anti-CD73 mAb with A2AR antagonist for therapy of patients with solid tumors are also ongoing in several phase I/II studies (NCT03274479, NCT03454451, NCT03549000, and NCT03381274).

**Table 1:** Current clinical trials based on NK cell checkpoint receptors or molecules.

**Checkpoint receptor**	**Ab**	**Combination**	**Malignancy**	**Phase**	**Status**	**ClinicalTrials.gov Identifier**
**KIR**	IPH4102		Cutaneous T-cell lymphoma	I	Active	NCT02593045
	Lirilumab	Nivolumab (anti-PD1 Ab)	Bladder cancer	I	Recruiting	NCT03532451
	Lirilumab	Nivolumab (anti-PD1 Ab)/Ipilimumab (anti-CTLA-4 Ab)	Advanced and/or metastatic solid tumors	I	Active	NCT03203876
	Lirilumab	Nivolumab (anti-PD1 Ab)	Advanced cancer	I	Recruiting	NCT03335540
	BMS-986015	Ipilimumab (anti-CTLA-4 Ab)	Advanced tumor	I	Completed	NCT01750580
	1-7F9		Multiple myeloma	I	Completed	NCT00552396
	Lirilumab	Nivolumab (anti-PD1 Ab)/Ipilimumab (anti-CTLA-4 Ab)	Advanced refractory solid tumors	I/II	Completed	NCT01714739
	Lirilumab	Nivolumab (anti-PD1 Ab)/Ipilimumab (anti-CTLA-4 Ab)	Solid tumors	I/II	Active	NCT03347123
	Lirilumab	Nivolumab (anti-PD1 Ab)	Multiple myeloma	I/II	Active	NCT01592370
	Lirilumab	Rituximab	Acute myeloid leukemia	I/II	Recruiting	NCT01256073
	Lirilumab	Nivolumab (anti-PD1 Ab)	Squamous cell carcinoma of the head and neck	II	Recruiting	NCT03341936
	Lirilumab	Rituximab (anti-CD20 Ab)	Lymphocytic leukemia	II	Completed	NCT02481297
	IPH2101		Smoldering multiple myeloma	II	Completed	NCT01222286
	IPH2101		Multiple myeloma	II	Completed	NCT00999830
	IPH4102	Gemcitabine and oxaliplatin	Advanced T cell lymphoma	II	Recruiting	NCT03902184
**NKG2A**	Monalizumab		Hematologic malignancies	I	Recruiting	NCT02921685
	Monalizumab		Gynecologic cancer	I	Completed	NCT02459301
	Monalizumab		Chronic lymphocytic leukemia	I/II	Terminated	NCT02557516
	Monalizumab	Cetuximab (anti-EGFR Ab)	Head and neck neoplasms	I/II	Recruiting	NCT02643550
	Monalizumab	Durvalumab (anti-PD-L1 Ab)	Advanced solid tumors	I/II	Active	NCT02671435
	Monalizumab		Stage III non-small cell lung cancer	II	Recruiting	NCT03833440
	Monalizumab	Durvalumab (anti-PD-L1 Ab)	Carcinoma/Squamous cell of head and neck	II	Recruiting	NCT03088059
**TIGIT**	OMP-313M32		Locally advanced/Metastatic cancer	I	Terminated	NCT03119428
	AB154	AB122(anti-PD1 Ab)	Advanced malignancies	I	Recruiting	NCT03628677
	MTIG7192A	Atezolizumab (anti-PD1 Ab)	Advanced/Metastatic tumors	I	Recruiting	NCT02794571
	MTIG7192A	Atezolizumab (anti-PD1 Ab)	Non-small cell lung cancer	II	Active	NCT03563716
**TIM-3**	Sym023		Metastatic/Solid tumor, lymphoma	I	Active	NCT03489343
	TSR-022		Advanced/Metastatic solid tumors	I	Recruiting	NCT02817633
	RO7121661		Solid tumors, metastatic melanoma, non-small cell lung cancer	I	Recruiting	NCT03708328
	LY3321367		Solid tumor	I	Active	NCT03099109
	MBG453	Spartalizumab (anti-PD1 Ab)	Glioblastoma multiforme	I	Active	NCT03961971
	MBG453		Acute myeloid leukemia	I	Recruiting	NCT03940352
	MBG453	PDR001 (anti-PD1 Ab)	Leukemia	I	Recruiting	NCT03066648
	INCAGN02390		Advanced malignancies	I	Recruiting	NCT03652077
	BGB-A425	Tislelizumab (anti-PD1 Ab)	Locally advanced/metastatic solid tumors	I/II	Recruiting	NCT03744468
	MBG453	PDR001 (anti-PD1 Ab)	Advanced malignancies	I/II	Recruiting	NCT02608268
	TSR-022	TSR-042 (anti-PD1 Ab)	Liver cancer	II	Recruiting	NCT03680508
**LAG-3**	Sym022		Metastatic cancer, solid tumor, lymphoma	I	Completed	NCT03489369
	TSR-033		Advanced solid tumors	I	Recruiting	NCT03250832
	BMS-986016	Nivolumab (anti-PD1 Ab)	Glioblastoma	I	Recruiting	NCT02658981
	BMS-986016	Nivolumab (anti-PD1 Ab)	Glioblastoma	I	Recruiting	NCT03493932
	REGN3767	REGN2810 (anti-PD1 Ab)	Malignancies	I	Recruiting	NCT03005782
	MGD013		Advanced solid tumors, hematologic neoplasms	I	Recruiting	NCT03219268
	Relatlimab	Nivolumab (anti-PD1 Ab)	Advanced solid tumors	I	Active	NCT02966548
	Relatlimab	Nivolumab (anti-PD1 Ab)	Gastric cancer, cancer of the stomach	I	Active	NCT03662659
	Relatlimab	Nivolumab (anti-PD1 Ab)/Carboplatin/ Paclitaxel/Radiation	Gastric/Esophageal Cancer	I	Recruiting	NCT03044613
	FS118		Advanced/Metastatic cancer	I	Active	NCT03440437
	IMP321		Solid tumors	I	Recruiting	NCT03252938
	IMP321		Renal cell carcinoma	I	Completed	NCT00351949
	IMP321		Metastatic breast cancer	I	Completed	NCT00349934
	IMP321	Pembrolizumab (anti-PD1 Ab)	Melanoma	I	Completed	NCT02676869
	BMS-986016		Hematologic neoplasms	I/II	Active	NCT02061761
	Relatlimab		Solid tumors	I/II	Recruiting	NCT01968109
	Relatlimab	Nivolumab (anti-PD1 Ab)	Various advanced cancer	I/II	Active	NCT02488759
	IMP321	Paclitaxel	Adenocarcinoma breast stage IV	II	Active	NCT02614833
	IMP321	Pembrolizumab (anti-PD1 Ab)	Non-small cell lung carcinoma, head and heck carcinoma	II	Recruiting	NCT03625323
	Relatlimab	Nivolumab (anti-PD1 Ab)	Melanoma	II	Recruiting	NCT03743766
	Relatlimab	Nivolumab (anti-PD1 Ab)	Advanced chordoma	II	Recruiting	NCT03623854
	Relatlimab	Nivolumab (anti-PD1 Ab)	Advanced cancer	II	Active	NCT02750514
	Relatlimab	Nivolumab (anti-PD1 Ab)	Advanced cancer	II	Recruiting	NCT02996110
	Relatlimab	Nivolumab (anti-PD1 Ab)	Advanced gastric cancer	II	Recruiting	NCT02935634
	Relatlimab	Nivolumab (anti-PD1 Ab)	Melanoma	II/III	Recruiting	NCT03470922
**PD-1**	Pembrolizumab		Advanced solid tumor	I	Recruiting	NCT03590054
	Pembrolizumab	IL-12	Metastatic solid tumor, Unresectable solid tumor	I	Recruiting	NCT03030378
	Pembrolizumab	BDB001 (TLR agonist)	Solid tumor	I	Recruiting	NCT03486301
	TSR-042		Neoplasms	I	Recruiting	NCT02715284
	Nivolumab Pembrolizumab	iPSC-derived NK Cells	Advanced solid tumors	I	Recruiting	NCT03841110
	Pembrolizumab	DC-NK Cells	Solid tumor	I	Not yet recruiting	NCT03815084
	Nivolumab	NK Cells	Renal cell carcinoma	I	Recruiting	NCT03891485
	Toripalimab		Solid tumors	I	Completed	NCT02857166
	Toripalimab		Advanced solid tumors	I	Enrolling by invitation	NCT03713905
	Toripalimab		Advanced malignancies	I	Recruiting	NCT03474640
	Toripalimab		Melanoma/Urological cancer	I	Active	NCT02836795
	JS001	Surufatinib (anti-VEGFR Ab)	Advanced solid tumors	I	Recruiting	NCT03879057
	HLX10		Solid tumor	I	Recruiting	NCT03468751
	LZM009		Solid tumor	I	Completed	NCT03286296
	GB226		Advanced and (or) recurrent solid tumor, lymphoma	I	Recruiting	NCT03374007
	INCMGA00012		Advanced solid tumors	I	Recruiting	NCT03059823
	AK105		Advanced cancer	I	Recruiting	NCT03352531
	SG001		Advanced solid tumors	I	Not yet recruiting	NCT03852823
	CS1003		Solid tumor, lymphoma	I	Recruiting	NCT03809767
	SCT-I10A		Solid tumor, lymphoma	I	Recruiting	NCT03821363
	Sym021	Sym022 (anti-LAG3 Ab)/Sym023 (anti-TIM3 Ab)	Advanced solid tumors, lymphomas	I	Recruiting	NCT03311412
	Nivolumab	BGB-290 (PARP inhibitor)	Advanced solid tumors	I	Active	NCT02660034
	Nivolumab	BGB-A1217 (anti-TIGIT Ab)	Advanced solid tumors	I	Recruiting	NCT04047862
	Nivolumab	RGX-104	Advanced solid malignancies, lymphoma	I	Recruiting	NCT02922764
	Avelumab	DC1c + myeloid DC + Ipilimumab	Neoplasms	I	Recruiting	NCT03707808
	ABBV-181	Rovalpituzumab (anti-delta-like 3 protein)/Venetoclax	Advanced solid tumors	I	Recruiting	NCT03000257
	Sintilimab	IBI310	Advanced solid tumors	I	Recruiting	NCT03545971
	SHR-1210	SHR3162 (Parp inhibitor)	Advanced solid tumors	I	Unknown	NCT03182673
	JNJ 63723283	anti-FGFR1-4	Neoplasms	I	Recruiting	NCT03547037
	Pembrolizumab	Allogeneic NK Cells	Biliary tract cancer	I/II	Recruiting	NCT03937895
	Pembrolizumab	Autologous DC-CIK cell	Advanced solid tumors	I/II	Recruiting	NCT03190811
	Pembrolizumab	Lenalidomide	Non-small cell lung cancer	I/II	Terminated	NCT02963610
	Pembrolizumab	MRx0518	Solid tumors	I/II	Recruiting	NCT03637803
	Pembrolizumab		lymphoma	I/II	Recruiting	NCT02535247
	Pembrolizumab	AGEN1884	Advanced solid cancers	I/II	Recruiting	NCT02694822
	Pembrolizumab	AST-008 (TLR9 agonist)	Advanced solid tumors	I/II	Recruiting	NCT03684785
	Pembrolizumab	Poly-ICLC	Metastatic colon Cancer	I/II	Recruiting	NCT02834052
	Tislelizumab	BGB-A333 (anti PD-L1 Ab)	Advanced solid tumors	I/II	Active	NCT03379259
	Nivolumab	Ipilimumab/BMS-986218	Advanced solid tumors	I/II	Recruiting	NCT03110107
	Nivolumab	BMS-986207 (anti-TIGIT Ab)	Advanced solid tumors	I/II	Recruiting	NCT02913313
	Nivolumab	Relatlimab/Ipilimumab + BMS-986205 (IDO1 inhibitor)	Advanced cancer	I/II	Recruiting	NCT03459222
	Nivolumab	Adenoviral product (Ad-p53) +pembrolizumab/capecitabine	Metastatic solid tumor, recurrent head and neck cancer	I/II	Recruiting	NCT02842125
	Nivolumab	RP1 (Genetically modified HSV for tumor lysis)	Melanoma/Bladder cancer, Non-melanoma skin cancer	I/II	Recruiting	NCT03767348
	PD-1 Ab	NK Cells	Non-small cell lung cancer	II	Recruiting	NCT03958097
	Avelumab	haNKTM	Merkel cell carcinoma	II	Recruiting	NCT03853317
	Pembrolizumab		Merkel cell carcinoma	II	Recruiting	NCT03241927
	Pembrolizumab		Advanced solid tumor	II	Recruiting	NCT02721732
	Pembrolizumab		Metastatic, recurrent or locally advanced cancer	II	Recruiting	NCT03428802
	Pembrolizumab		Advanced non-small cell lung carcinoma	II	Recruiting	NCT03447678
	Pembrolizumab	Nab-paclitaxel + anti-cancer drugs	Malignant Neoplasm of Breast	II	Active	NCT03289819
	HX008		Advanced solid tumors	II	Recruiting	NCT03704246
	Tislelizumab		MSI-H/dMMR solid tumors	II	Recruiting	NCT03736889
	Nivolumab	Metformin/Rosiglitazone	Solid tumor malignancies	II	Not yet recruiting	NCT04114136
	Nivolumab	Ipilimumab	Advanced solid tumors	II	Recruiting	NCT02834013
	Nivolumab	Axitinib (anti-VEGF Ab)	Renal cell carcinoma	II	Recruiting	NCT03595124
	Nivolumab	Relatlimab (anti-LAG-3 Ab)Ipilimumab (anti-CTLA-4 Ab)	Head and neck cancer	II	Recruiting	NCT04080804
	Nivolumab	Pembrolizumab + trigriluzole (PKC inhibitor)	Metastatic/Unresectable malignancies, lymphoma	II	Recruiting	NCT03229278
	AGEN2034	AGEN1884	Cervical cancer	II	Recruiting	NCT03894215
**PD-L1**	HLX20		Advanced solid tumors	I	Recruiting	NCT03588650
	KN035		Advanced/Metastatic solid tumors	I	Unknown	NCT03248843
	KN035		Hepatocellular carcinoma	I	Unknown	NCT03101488
	Atezolizumab		Neoplasms	I	Recruiting	NCT02862275
	CS1001		Advanced solid tumors	Ia/Ib	Recruiting	NCT03312842
	MSB2311		Advanced solid tumors	I	Recruiting	NCT03463473
	SHR-1316		Advanced solid tumors	I	Completed	NCT03133247
	CK-301		Advanced cancers	I	Recruiting	NCT03212404
	LY3300054	Prexasertib (CHK1 inhibitor)	Advanced solid tumors	I	Active	NCT03495323
	Durvalumab	Tremelimumab + stereotactic body radiotherapy	Non-small cell lung cancer	Ib	Recruiting	NCT03275597
	Durvalumab	Tremelimumab +metronomic vinorelbine	Advanced solid tumors	I/II	Suspended	NCT03518606
	Durvalumab	Tremelimumab + azacitidine	Head and neck cancer	I/II	Recruiting	NCT03019003
	Avelumab	Utomilumab + PF-04518600 + radiation therapy	Advanced malignancies	I/II	Recruiting	NCT03217747
	Durvalumab	Tremelimumab + fulvestrant	Breast cancer	II	Unknown	NCT03430466
	Durvalumab	Tremelimumab + radiation therapy	Bladder cancer	II	Recruiting	NCT03601455
	Durvalumab	Tremelimumab	Advanced solid tumors	III	Active	NCT03084471
**CD73/A2AR**	Ciforadenant	CPI-006	Advanced cancers	I	Recruiting	NCT03454451
	MEDI9447	MEDI4736	Solid tumors	I	Active	NCT02503774
	Ciforadenant	Atezolizumab	Renal cell cancer, Metastatic castration resistant prostate cancer	I	Recruiting	NCT02655822
	Ciforadenant AZD4635	Durvalumab (anti-PD-1 Ab)	Advanced solid malignancies	I	Recruiting	NCT02740985
	NZV930 NIR178	PDR001	Advanced malignancies	I	Recruiting	NCT03549000
	PBF-509:	PDR001	Non-small cell lung cancer	I/II	Recruiting	NCT02403193
	Oleclumab AZD4635	Osimertinib	Non-small cell lung cancer	I/II	Active	NCT03381274
	NIR178	PDR001	Solid tumors, Non-Hodgkin lymphoma	II	Recruiting	NCT03207867

## Combination Therapies Comprising Different Checkpoint Receptor Blockades or Combined With Other Therapeutic Strategies

Although therapy with checkpoint inhibitors, particularly anti-PD-1/PD-L1 and anti-CTLA-4 mAbs, has demonstrated marked success in recovering anti-tumor immune responses and improving the survival of patients with advanced malignancy, the response rates are only approximately 20 to 40% using given a checkpoint inhibitor alone (122). Many patients do not respond or develop resistance to these therapeutic approaches (134). It is likely that interrupting a single checkpoint is not enough to release the dysfunction of NK or T cells, and some cancers develop primary resistance or may adapt to acquire resistance to further therapies (134). Combination therapies with other immune checkpoint inhibitors are currently being investigated to overcome this resistance. Increasing evidence indicates the promise of this approach by enhancing anti-tumor efficacy and increasing the response rates (135). Exploiting the different mechanisms that impair T cell response, co-blockade of CTLA-4 and PD-1 (Ipilimumab + Nivolumab) revealed additional anti-tumor efficacy but also high levels of side effects in treatment of melanoma (136). A newly report of the 5-year outcome of combination therapy with Nivolumab and Ipilimumab in advanced melanoma has shown encouraging results (137). At a minimum follow-up of 60 months, the median overall survival is more than 60 months in Ipilimumab + Nivolumab group, 36.9 months in the nivolumab group, and 19.9 months in the ipilimumab group. The 5-year overall survival rate is 52% in the Ipilimumab + Nivolumab group, as compared with 44% in the nivolumab group and 26% in the ipilimumab group. These results demonstrate that the combination of anti-PD-1 mAb and anti-CTLA-4 mAb can prolong the response rate and overall survival of patients with advanced melanoma, supporting the promise of combination approach. Other combinations of checkpoint blockades, such as anti-PD-1 or PD-L1 mAbs in combination with Abs targeting TIM-3, LAG-3, or TIGIT, are also being assessed in ongoing clinical trials (Table 1) or are under investigation.

Notably, the common reasons leading to tumor resistance to checkpoint inhibitors include low antigenicity and the defects in the presentation of tumor-cell antigens (e.g., mutation or deficiency in MHC molecules), which are necessary to prime anti-tumor T cell immunity (134, 138). In these circumstances, NK cells can play critical anti-tumor role because the activity of NK cells is not limited by antigenicity and lacks MHC restriction. Consequently, it is critical to recover the NK cell's anti-tumor function by blockade of NK cell-specific checkpoint receptors or using combination blockade with NK cell checkpoint receptor and monoclonal antibodies recognizing CTLA-4 or PD-1/PD-L1. More importantly, activated NK cells can further promote T cell priming and activation, thus leading to enhanced innate and adaptive anti-tumor activity. Recent research reported that inhibitory KIRs and PD-1 correlated positively in NSCLC, suggesting that a combination of anti-KIR mAbs and anti-PD-1 mAbs could be a critical therapeutic strategy for patients with NSCLC (139). Currently, phase I/II clinical trials of a combination of anti-KIR mAbs and anti-PD-1 or anti-CTLA-4 mAbs have finished and indicated that the therapy was safe and effective for patients with advanced solid tumors (NCT01750580, NCT01714739). Indeed, targeting NK cell checkpoint receptors using anti-TIGIT and anti-NKG2A mAbs could restore the anti-tumor efficacy of both NK and T cells, and more significantly, that anti-tumor capacity could be amplified using a combination with anti-PD-1/PD-L1 mAbs in various tumor models (6, 15). A phase II clinical trial (NCT02671435) of the combination of anti-NKG2A mAbs and anti-PD-1 mAbs showed encouraging results for patients with CRC, particularly those resistance to therapy with anti-PD-1/PD-L1 mAbs (113). Recently, combinations of tumor antigen-targeted IgG Abs with checkpoint inhibitors have displayed promising results. One of the most critical effects of IgG Abs is to induce NK cell-mediated ADCC. Accordingly, the combination of checkpoint inhibitors with IgG mAbs was observed to enhance innate and adaptive anti-tumor capacity, thereby increasing the efficacy of tumor elimination. The combination of monalizumab and cetuximab to treat patients with SCCHN has yielded an encouraging outcome with an improved response rate (NCT02643550) (15). Currently, checkpoint inhibitors in combination with mAbs targeting HER-2, EGFR, and CD20 have been evaluated in pre-clinical and clinical trials for the treatment of various types of tumors, such as CRC, HNSCC, non-Hodgkin's lymphoma, and CLL (140).

Combining checkpoint inhibitors with conventional therapy (e.g., radiation therapy, targeted therapy, and chemotherapy) or other immunotherapies are also being investigated in clinical trials and/or animal models. Low-dosage chemotherapies plus checkpoint blockers appeared promising in experimental animal models and clinical studies. A phase III clinical trial applying anti-PD-1 mAbs (Pembrolizumab) in combination with chemotherapy to treat metastatic NSCLC has demonstrated markedly increased overall survival compared with that of chemotherapy alone (NCT02578680) (141, 142). Low-dose decitabine is reported to increase the expression of MHC and cytokines, and improve lymphocyte infiltration into tumor sites, thereby enhancing the efficiency of PD-1 blockade in CRC with microsatellite stability by re-modulating the TME (143). Radiation therapy can upregulate PD-1/PD-L1 expression on tumor cells and immune cells, thus increasing sensitivity to therapy with PD-1/PD-L1 inhibitors. In particular, radiation therapy can promote NK cells long-term migration and infiltration into tumor sites, and the long-term survival of NK cells (144). Certain ongoing and completed clinical trials of the combination of checkpoint blockade with chemotherapy or radiation therapy to treat different types of tumors have shown beneficial effect (145). A number of studies or clinical trials investigating checkpoint inhibitors combined with targeted therapies (e.g., anti-angiogenic therapy, B-RAF and MEK inhibitors, and CDK4/6 inhibitors), epigenetic modulators, and TLR agonists are currently being carried out in various types of cancers. For instance, a large phase III clinical trial with anti-PD-L1 mAb plus anti-VEGF mAb (bevacizumab) has recently confirmed that the combination therapy can extend the progression-free survival of patients with previously untreated metastatic RCC when compared to therapy with sunitinib (146).

It is noteworthy that the NK cell frequency appeared to correlate with responsiveness to checkpoint blockade therapies and increased survival in various types of cancers (147). In patients with melanoma at different stages of treatment, a high frequency of tumor-infiltrating NK cells predicted their responsiveness to anti-PD-1 mAb therapy (148). Not only checkpoint inhibitor therapies promote the direct anti-tumor efficacy of NK cells, but also, NK cells can promote stimulatory DCs recruitment to tumor sites by secreting cytokine Fms-related tyrosine kinase 3 ligand (FLT3LG), which subsequently enhances the responses of patients to anti-PD-1 therapy and promotes increased overall survival (148, 149). Accordingly, intervention strategies combining the recruitment and activation of NK cells (such as cytokines, TLR agonists, STING agonists) suggest great promise to improve the responsiveness and outcomes of checkpoint blockade therapy. For example, the combination of anti-PD-1 and/or anti-LAG-3 mAbs with IL-12, which promotes NK cell recruitment and activation, significantly enhanced the anti-metastatic effect that entirely depended on NK cells in breast cancer (95). Pre-treatment with IFN-γ, the TLR3 ligand poly (I:C), and anti-IL-10 Ab, which attract IFN-γ-producing NK cell infiltration into tumor sites, rendered tumors sensitive to checkpoint blockade therapy (e.g., mAbs recognizing CTLA-4 and PD-1), leading to increased cure rates. Depletion of NK cells abrogated this sensitizing effect (150). Currently, there are several ongoing clinical trials investigating polyICLC, a stable derivative of poly (I:C), combined with checkpoint inhibitors (NCT02643303, NCT02834052, NCT03721679). In addition, combining checkpoint blockade with the transfer of activated NK cells or CAR-engineered NK cells is also a beneficial approach to improve the response rates and therapeutic efficacy of immune checkpoint therapy.

## Perspectives

**Figure 1:**
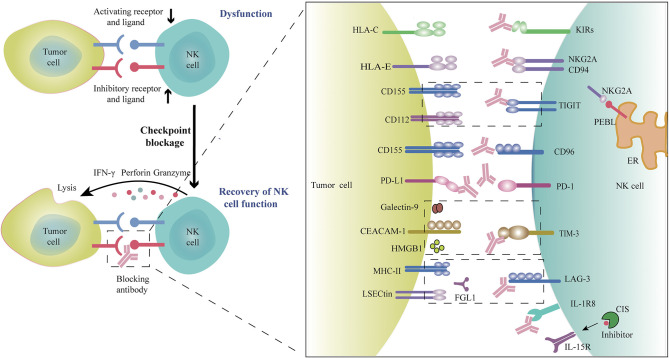
Blockade of checkpoint inhibitors or molecules promotes NK cell anti-tumor efficacy. Many factors in the tumor microenvironment induce NK cell dysfunction, as evidenced by the upregulation of inhibitory checkpoint receptors and the downregulation of activating receptors on tumor-infiltrating NK cells, which can result in NK cell exhaustion. Blocking these immune checkpoint receptors or molecules using monoclonal antibodies would restore the anti-tumor activity of NK cells. Rationally, co-combination with different checkpoint inhibitors or combinations of checkpoint inhibitors with other therapeutic agents could further amplify anti-tumor efficacy.

Recently, immune checkpoint blockade, particularly using mAbs targeting PD-L1, PD-1, or CTLA-4 has achieved outstanding therapeutic efficacy for patients with metastatic melanoma, NSCLC, microsatellite stable colorectal cancer, and other tumor types. These therapies rely mainly on generating robust T cell anti-tumor immune responses. Increasing evidence shows that NK cell-based therapy is an effective supplement to T-cell therapy in that they have unique advantages over conventional T cells in certain respects. Blockade of NK cell-specific checkpoint receptors to rescue TME-induced dysfunction of NK cells (Figure 1) can reinvigorate NK cells' direct cytotoxic activity against tumors and further prime and enhance the T cell-mediated adaptive anti-tumor immunity, especially for those low antigenicity and MHC^neg^ tumors that are resistant to T cell recognition and cytolysis. Although the preliminary data from clinical trials indicate that blockade of NK cell-specific checkpoints seems not as efficient as anti-PD-1 mAbs, combination with other checkpoint inhibitors or other therapeutics has shown great promise in clinical trials and pre-clinical studies. In particular, the combination of anti-NKG2A mAbs or anti-TIGIT mAbs plus anti-PD-1 mAbs or cetuximab have demonstrated encouraging outcome in the treatment of patients with advanced solid tumors (6, 15). Other combination strategies are under investigation or in clinical trials, such as combining checkpoint inhibitors with agents that promote NK cell infiltration into tumors, promote the persistence and activation of NK cells (such as cytokines and TLR or STING agonists), and resist the inhibitory TME (e.g., anti-TGF-β mAbs), to further increase anti-tumor capacity of NK cells and improve the response rates to immunotherapies. A combination of NK checkpoint inhibition with adoptive transfer of CAR-engineered NK cells or allogeneic activated NK cells would seem a reasonable approach. Specific stimulation or induction of the anti-tumor capacity of certain NK cell subpopulations, such as memory-like or adaptive NK cells, might achieve more powerful efficacy and better clinical outcomes. Recently, increasing evidence has highlighted the crucial role of the cellular metabolic program in directing the development, survival, proliferation, and function of immune cells, and tumors and metabolic products in the TME induce the dysfunction and exhaustion of T and NK cells by modulating cell metabolism (40, 151–154). Novel strategies might emerge from increased knowledge of the metabolic requirements of activated NK cells through targeting metabolic reprogramming and related pathways or factors to improve NK cell function more effectively within the TME (155). TLR signaling is involved in the metabolic reprogramming of the TME, thus regulating T cell function. Therefore, TLR agonists combined with checkpoint inhibitors and/or adoptive T cell therapy is proposed as a novel strategy for cancer immunotherapy (156). Similar strategies are also appropriate for NK cell-based immunotherapy. The tumor heterogeneity and the complex TME across different patients and tumor types form major barriers to anti-tumor responses and impair the responsiveness of patients to specific checkpoint blockade therapy. A deeper understanding of the immune phenotypes of tumors, lymphocyte infiltration, and the TME composition, are important to identify appropriate patient populations to receive specific therapies and could help to prioritize the most effective therapies or therapeutic combinations. It is crucial to identify suitable biomarkers to predict and monitor the response to specific therapy, appropriate therapeutic combinations, and therapeutic efficacy (157–159). Personalized therapy based on particular patients is a future goal.

## Author Contributions

CZ designed and wrote the manuscript. YL searched the literature and drafted the figures. All authors revised and approved the final version of the manuscript.

## Conflict of Interest

The authors declare that the research was conducted in the absence of any commercial or financial relationships that could be construed as a potential conflict of interest.
